# Intratracheal administration of mesenchymal stem cells modulates lung macrophage polarization and exerts anti-asthmatic effects

**DOI:** 10.1038/s41598-022-14846-y

**Published:** 2022-07-11

**Authors:** Yosep Mo, Hanbit Kang, Ji-Young Bang, Jae Woo Shin, Hye Young Kim, Sang-Heon Cho, Hye-Ryun Kang

**Affiliations:** 1 Institute of Allergy and Clinical Immunology, Seoul National University Medical Research Center, 103 Daehak-ro, Jongno-gu, Seoul, 03080 Korea; 2 Department of Translational Medicine, Seoul National University College of Medicine, 103 Daehak-ro, Jongno-gu, Seoul, 03080 Korea; 3 Department of Medical Science, Seoul National University College of Medicine, 103 Daehak-ro, Jongno-gu, Seoul, 03080 Korea; 4 Department of Internal Medicine, Seoul National University College of Medicine, 103 Daehak-ro, Jongno-gu, Seoul, 03080 Korea

**Keywords:** Adaptive immunity, Inflammation, Innate immune cells, Innate immunity, Immunology, Stem cells, Diseases

## Abstract

Mesenchymal stem cells (MSCs) possess immunomodulatory properties that have therapeutic potential for the treatment of inflammatory diseases. This study investigates the effects of direct MSC administration on asthmatic airways. Umbilical cord MSCs (ucMSCs) were intratracheally administered to six-week-old female BALB/c mice sensitized and challenged with ovalbumin; airway hyperresponsiveness (AHR), analyses of airway inflammatory cells, lung histology, flow cytometry, and quantitative real-time PCR were performed. Furthermore, ex vivo and in vitro experiments were performed to assess the effects of ucMSC on M2 activation. Intratracheally administered ucMSCs decreased degree of airway resistance and the number of inflammatory cells such as T helper 2 (Th2) cells, type 2 innate lymphoid cells (ILC2), and macrophages in the murine asthma model. Particularly, MHCII and CD86 expression diminished in dendritic cells and alveolar macrophages (AMs) following ucMSC treatment. SiglecF^+^CD11c^+^CD11b^-^ AMs show a negative correlation with type II inflammatory cells including Th2 cells, ILC2, and eosinophils in asthmatic mice and were restored following intratracheal ucMSCs treatment. In addition, ucMSCs decreased the macrophage polarization to M2, particularly M2a. The expression levels of markers associated with M2 polarization and Th2 inflammation were also decreased. ucMSC reduced *Il-12* and *Tnfa* expression as well as that of M2 markers such as *Cd206* and *Retnla *ex vivo. Furthermore, the in vitro study using IL-4 treated macrophages confirmed that both direct and indirect MSC treatment significantly reduced the expression of *Il-5* and *Il-13*. In conclusion, ucMSCs appear to suppress type II inflammation by regulating lung macrophages via soluble mediators.

## Introduction

Asthma is a chronic inflammatory disease characterized by airway hyperresponsiveness (AHR), airway obstruction and remodeling. Allergic asthma is typically characterized by T helper 2 (Th2) inflammation which usually shows a good response to inhaled corticosteroids^1^. However, symptoms of severe asthma are sometimes refractory to high-dose inhaled corticosteroids which shows a need for alternative methods of anti-inflammatory treatment^2^.

Mesenchymal stem cells (MSCs), such as bone marrow-derived MSCs (bmMSCs), umbilical cord-derived MSCs (ucMSCs), and adipose tissue-derived MSCs (ASC), are progenitor cells with multipotent, non-hematopoietic, self-renewing abilities. Recently, it was discovered that MSCs act as immune modulators and affect various immune cells by inhibiting the proliferation of CD4^+^ and CD8^+^ T cells, modulating the plasticity of the role of T cells, including regulatory T cells (Tregs)^3–5^, and regulating the landscape of circulating monocytes, macrophages and dendritic cells (DCs). In addition, primed or licensed MSCs have reduced or amplified immunomodulatory capacity, depending on the environment of the inflammatory mediators, including interferon-γ (IFN-γ)^6^. Recently, it was found that licensed MSCs play an immunomodulatory role by regulating macrophage activation^7^. However, although MSCs play a role in macrophage polarization, the mechanism of this process in response to MSC treatment in asthma is yet to be elucidated^8^.

Macrophages are the most common type of immune cells in the airway, and these cells are able to differentiate into M1 or M2 subtypes upon stimulation^9^. M1 macrophages are induced by Th1 stimulation via IFN-γ, and are characterized as pro-inflammatory macrophages, In contrast, M2 macrophages are induced by Th2 stimulation via interleukin (IL)-4 and IL-13, and are characterized as having anti-inflammatory and repairing properties^10^. Recent studies with murine asthma models suggest a possible relationship between the M2 macrophage subtypes and asthma pathogenesis^11^. In particular, it determines landscape for the development of allergic asthma combined with Th2 cells, however, therapeutic approach targeting M2 macrophage subtypes have not been clarified^12,13^.

This study investigates the immunomodulatory effects of intratracheally administered ucMSCs in an ovalbumin (OVA)-induced murine asthma model. In addition, the effects of ucMSCs on macrophage subtype differentiation and innate immune cell activation are also investigated.

## Results

### Reduction of AHR and inflammation by ucMSC treatment in a murine asthma model

**Figure 1:**
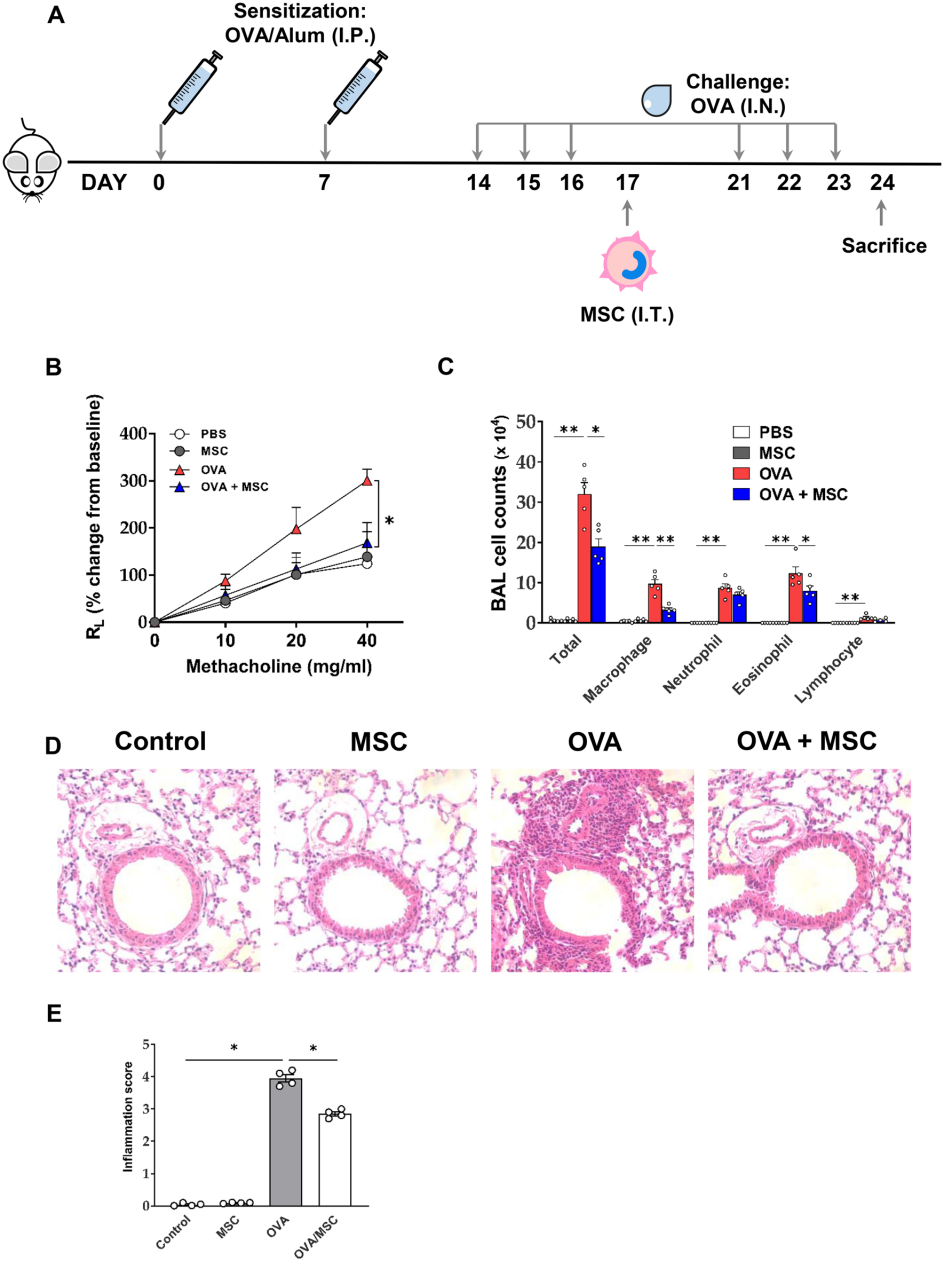
Effect of ucMSC on airway hyperresponsiveness and lung inflammation in a murine asthma model. (**A**) To establish a murine asthma model, 2 mg of Alum and 100 μg of OVA in 100 μL of PBS were injected into the mouse in the peritoneum on days 0 and 7, and 50 μg of OVA in 40 μL of PBS was administered intranasally on days 14, 15, and 16. ucMSCs (10^5^ cells) were injected intratracheally on day 17. (**B**) Methacholine hyperresponsiveness was measured 24 h after the last intranasal challenge. (**C**) The number of total inflammatory cells, including macrophages, neutrophils, eosinophils, and lymphocytes in BAL fluid. (**D**) H&E stain (× 100) of lung histology after allergen challenge. (E) Lung inflammation score in murine asthma model. (i: PBS, ii: ucMSC, iii: OVA, iv: OVA + ucMSC group). *n* = 4–5 for each group, * indicates *P* < 0.05, ** indicates *P* < 0.01. All results are representative of at least three independent experiments. OVA, ovalbumin; Alum, aluminum hydroxide; IP, intraperitoneal; IN, intranasal; MSC, mesenchymal stem cell, R_L_, resistance of lung; BAL, bronchoavleolar lavage.

To determine the therapeutic effect of human ucMSCs on asthmatic airways, an OVA-induced murine asthma model was intratracheally treated with ucMSCs. ucMSC treatment significantly decreased AHR and airway inflammation observed in the asthma model (Fig. 1B,C). Immune cell infiltration in the bronchi and the blood vessels was reduced in the histological analysis of the OVA + ucMSC group (Fig. 1D). In addition, histologic quantification scores of inflammatory cell infiltration in the tissues were significantly reduced after intratracheal ucMSC treatment (Fig. 1E). In addition, flow cytometry analysis showed that the number of eosinophils in the lungs was significantly decreased in the OVA + ucMSC group, whereas the number of neutrophils did not change in the OVA + ucMSC group (Fig. S3).

### Reduction of Th2 and ILC2 by ucMSC treatment in a murine asthma model

**Figure 2:**
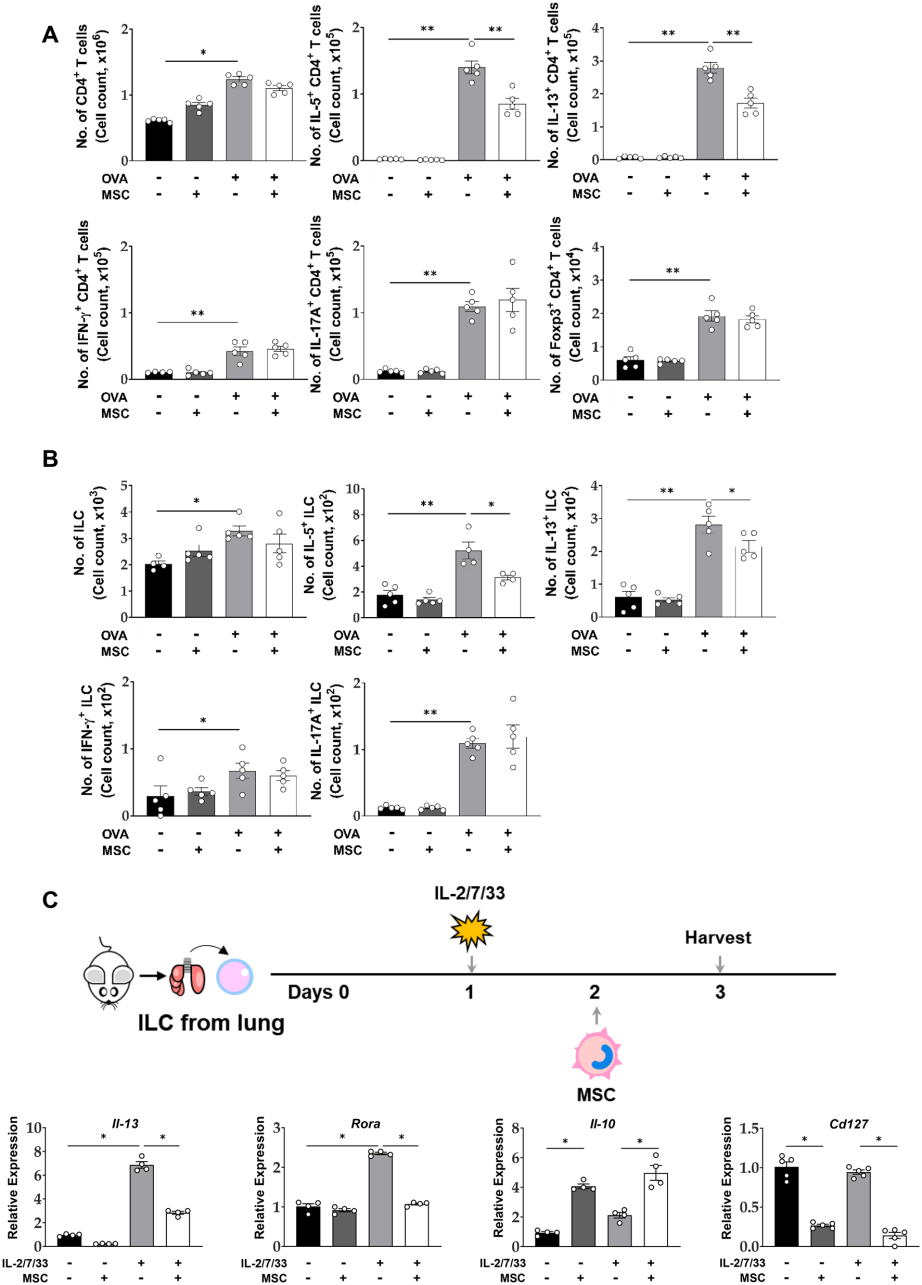
Effect of ucMSCs on Th2 cells and ILC2s by ucMSC treatment in a murine asthma model. (**A**) The number of lung CD4^+^ T cells, IL-5^+ ^CD4^+^ T cells, IL-13^+ ^CD4^+^ T cells, IFN-γ^+^ CD4^+^ T cells, IL-17A^+ ^CD4^+^ T cells and Foxp3^+ ^CD4^+^ T cells. (**B**) The number of lung ILCs, IL-5^+^ ILCs, IL-13^+^ ILCs, IFN-γ^+^ ILCs, and IL-17A^+^ ILCs. (**C**) ILC2s and ucMSCs co-culture experiment protocol and ILC2 activation, surface marker and *Il-10* expression. n = 4–5 for each group, * indicates *P* < 0.05, ** indicates *P* < 0.01. All results are representative of at least three independent experiments. MSC, mesenchymal stem cell; ILC, innate lymphoid cell; IL, interleukin; IFN, interferon.

The total number of CD4^+^ T cells and IL-5/IL-13-secreting CD4^+^ T cells was significantly increased in the OVA group. While intratracheal ucMSC treatment decreased the number of IL-5- or IL-13-secreting CD4^+^ T cells (Fig. 2A), no significant changes were observed in IFN-γ- or IL-17A-secreting CD4^+^ T cells and Treg cells (Fig. 2A).

The total number of innate lymphoid cells (ILCs) and IL-5/IL-13-secreting type 2 ILCs (ILC2s) was increased in the OVA group, and ucMSC treatment significantly reduced the total number ILCs and ILC2s. Similar to the results observed in Th1 and Th17 cells, intratracheal ucMSC treatment did not change the number of IFN-γ-secreting ILC1s and IL-17A-secreting ILC3s increased in the OVA group (Fig. 2B).

Next, we determined whether MSCs play an immunomodulatory role on ILC2. ucMSC inhibited the levels of IL-13 and master regulators of ILC2s. Interestingly, ucMSC treatment significantly increased *Il-10* expression and suppressed the expression of surface receptors (Fig. 2C).

### Changes in DCs and macrophages by ucMSC treatment in a murine asthma model

**Figure 3:**
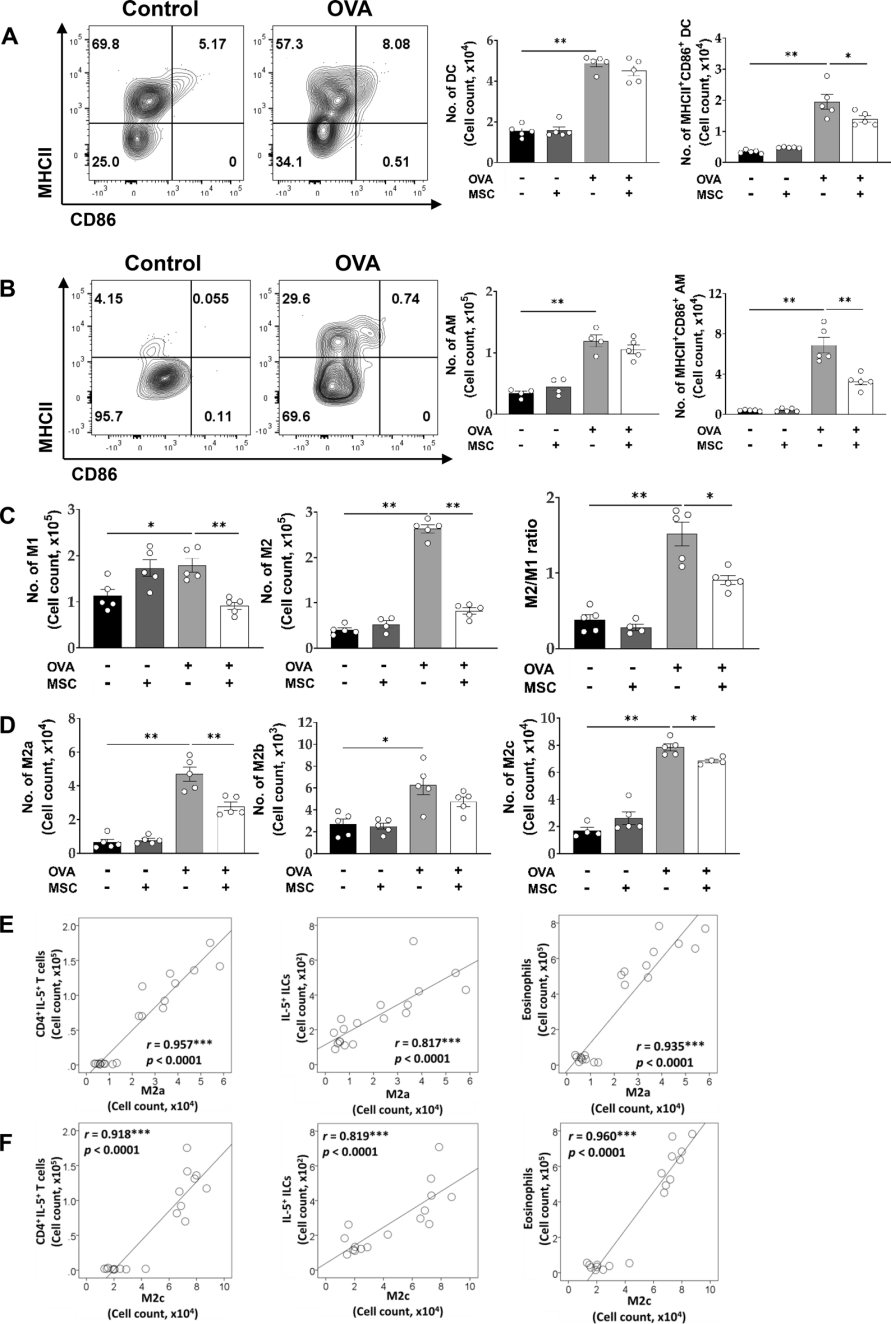
Effect of ucMSCs on DCs and macrophages by ucMSC treatment in a murine asthma model. (**A**) The number of lung DC, MHCII^+^CD86^+^ DC. (**B**) AM, MHCII^+^CD86^+^ AM. (**C**) The number of lung M1, M2, and DN and the ratio of M2/M1. (**D**) The number of M2a, M2b, and M2c macrophages. (**E**) Correlation plots between M2a macrophages and CD4^+^IL-5^+^ T cells, IL-5^+ ^ILCs, and eosinophils. (F) Correlation plots between M2c macrophages and CD4^+^ IL-5^+^ T cells, IL-5^+ ^ILCs, and eosinophils. *n* = 4–5 for each group, * indicates *P* < 0.05, ** indicates *P* < 0.01, *** indicates *P* < 0.0001. All results are representative of at least three independent experiments. MSC, mesenchymal stem cell; DC, dendritic cells; AM, alveolar macrophage; IL, interleukin; ILC, innate lymphoid cell.

The OVA challenge increased the number of DCs in the lung, especially those with enhanced expression of MHCII and CD86. Although ucMSC treatment did not reduce the total number of DCs, the number of mature DCs (MHCII^+^CD86^+^DCs) was significantly reduced with ucMSC treatment (Fig. 3A). ucMSC treatment also reduced the number of MHCII^+^CD86^+^ alveolar macrophages (AMs) in the OVA group (Fig. 3B).

The OVA challenge increased both M1 and M2 macrophages, and both subtypes were decreased after ucMSC treatment. Considering that the M2/M1 ratio was significantly decreased in the OVA + ucMSC group, M2 macrophages were more effectively reduced by ucMSCs (Fig. 3C).

Among the M2 subtypes, ucMSC treatment significantly reduced M2a and M2c populations; these populations showed strong positive correlations with type II immune cells such as IL-5- or IL-13-secreting CD4^+ ^T cells, IL-5- or IL-13-secreting ILC2s, and eosinophils (Fig. 3E,F and S4A, B). In contrast, the upregulated M2b population did not change after ucMSC treatment (Fig. 3D).

**Figure 4:**
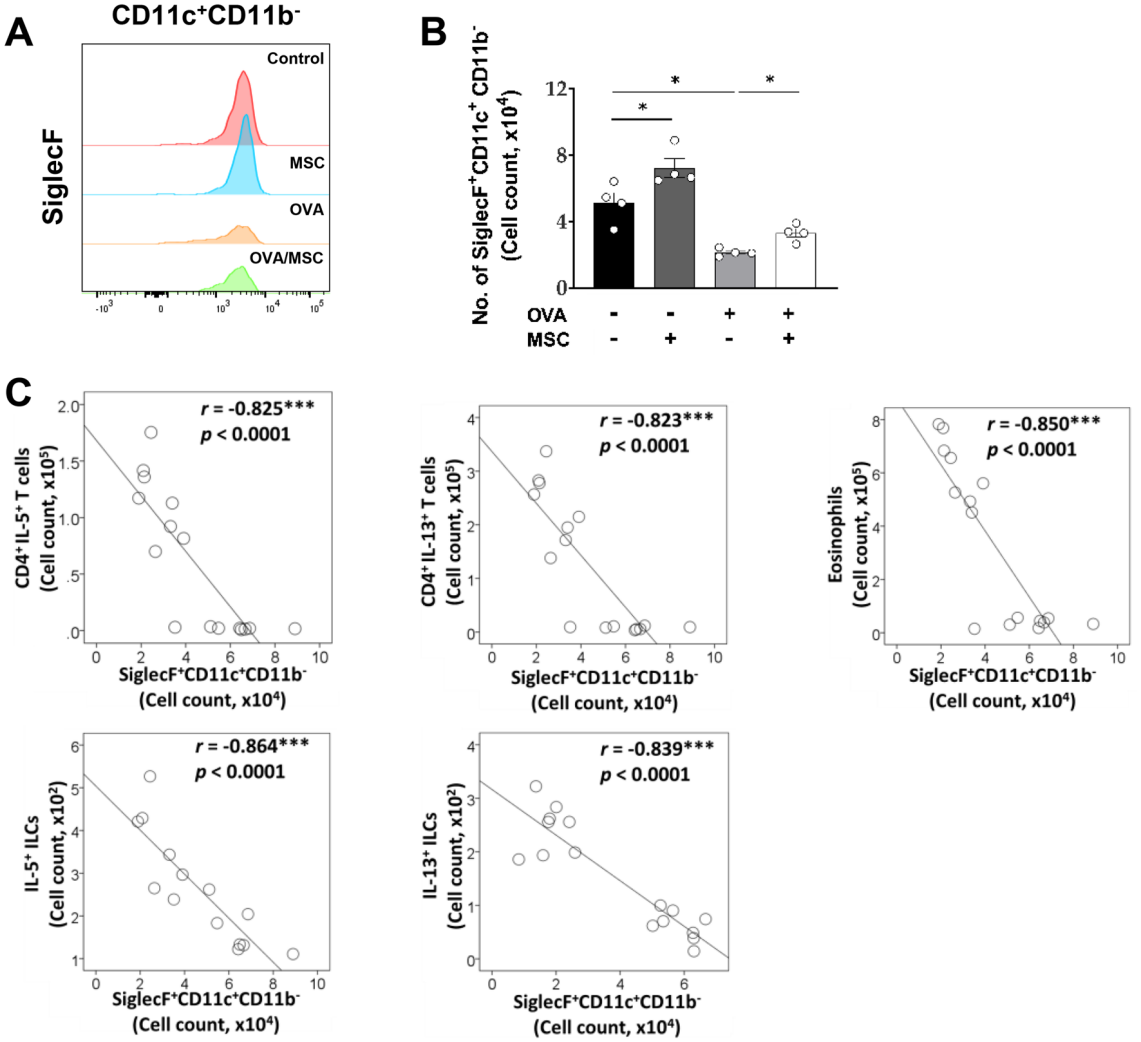
Effect of ucMSCs on CD11c^+^CD11b^-^ macrophages in a murine asthma model. (**A**) Expression level and MFI of SiglecF on CD11c^+^CD11b^-^ macrophages. (**B**) The number of SiglecF^+^CD11c^+^CD11b^-^ macrophages. (**C**) Correlation plots between SiglecF^+^CD11c^+^CD11b^-^ macrophages and CD4^+^IL-5^+^ T cells, CD4^+^IL-13^+^ T cells, eosinophils, IL-5^+^ ILCs, and IL-13^+^ ILCs. *n* = 4, * indicates *P* < 0.05, *** indicates *P* < 0.0001. All results are representative of at least three independent experiments. MSC, mesenchymal stem cell; IL, interleukin; ILC, innate lymphoid cell.

Through analysis of F4/80^+^ macrophages based on CD11c and CD11b, we classified these cells into different clusters: CD11c^+^CD11b^-^, CD11c^+^CD11b^+^, CD11c^-^CD11b^+^, and CD11c^-^CD11b^−^ (Fig. S5). SiglecF was highly expressed in CD11c^+^CD11b^-^ macrophages (Fig. 4A), and the SiglecF^+^CD11c^+^CD11b^-^ population was regarded as resident AMs. The proportion of these resident AMs was significantly reduced in the OVA-treated groups and restored upon ucMSC treatment (Fig. 4B). SiglecF^+^CD11c^+^CD11b^-^ macrophages showed strong negative correlations with type 2 immune cells (Fig. 4C).

### Changes in mRNA expression by ucMSC treatment in in vivo, ex vivo, and in vitro experiments

**Figure 5:**
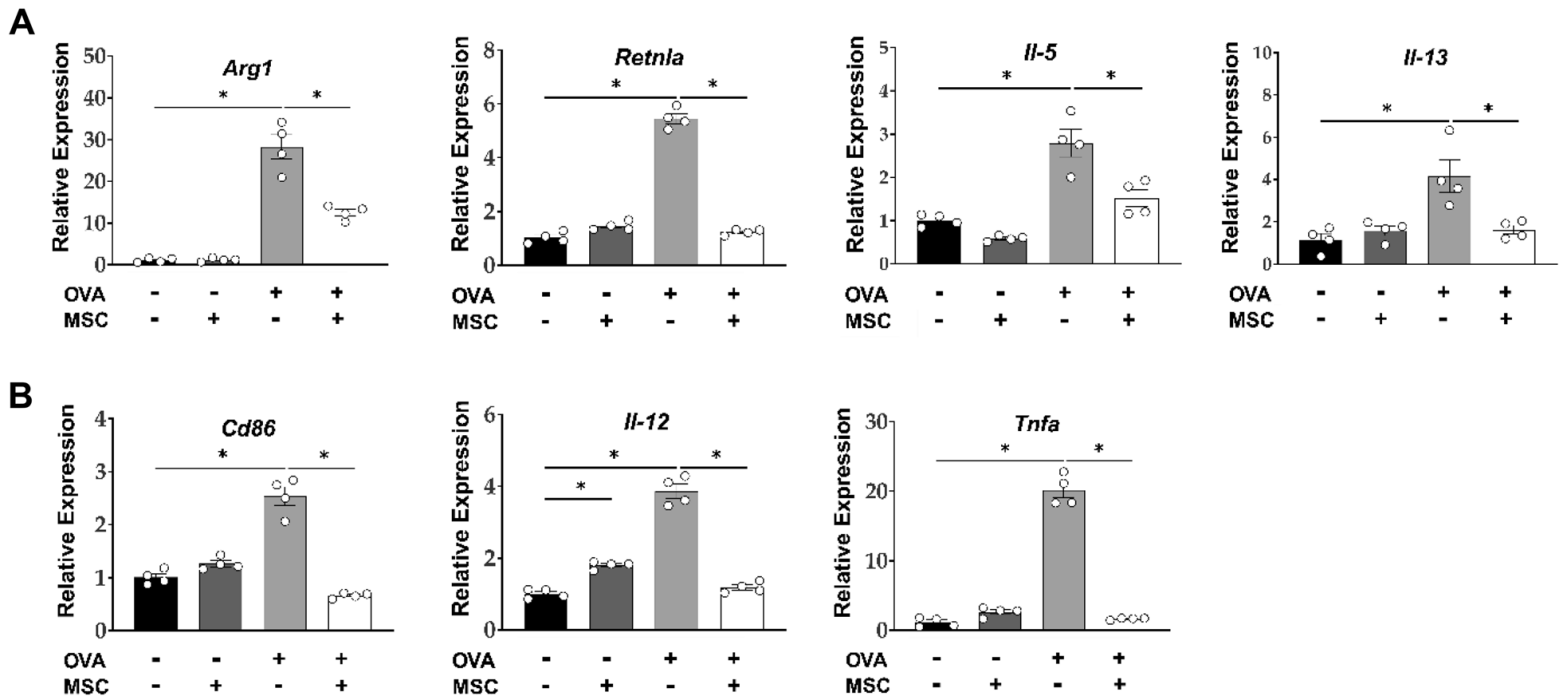
Effect of ucMSCs on mRNA expression of lung in a murine asthma model. (**A**, **B**) Changes in lung macrophage mRNA expression of (**A**) *Arg1*, *Retnlα, IL-5, Il-13.* (**B**) *Cd86, Il-12*, and *Tnfa*. *n* = 4 for each group, * indicates *P* < 0.05. All results are representative of at least three independent experiments. MSC, mesenchymal stem cell; IL, interleukin; TNF: tumor necrosis factor.

Analysis of mRNA expression levels in mouse lung homogenates confirmed that ucMSC treatment downregulated the expression levels of both M1 and M2 markers previously upregulated by the OVA challenge. Enhanced expression of M2 markers (*Arg1* and *Retnlα*) and Th2 cytokines (*Il-5* and *Il-13*) in the OVA group were also significantly reduced after ucMSC treatment (Fig. 5A). Similarly, ucMSCs downregulated M1 markers including *CD86, Il-12,* and *Tnfa* that had been upregulated by OVA challenge (Fig. 5B).

**Figure 6:**
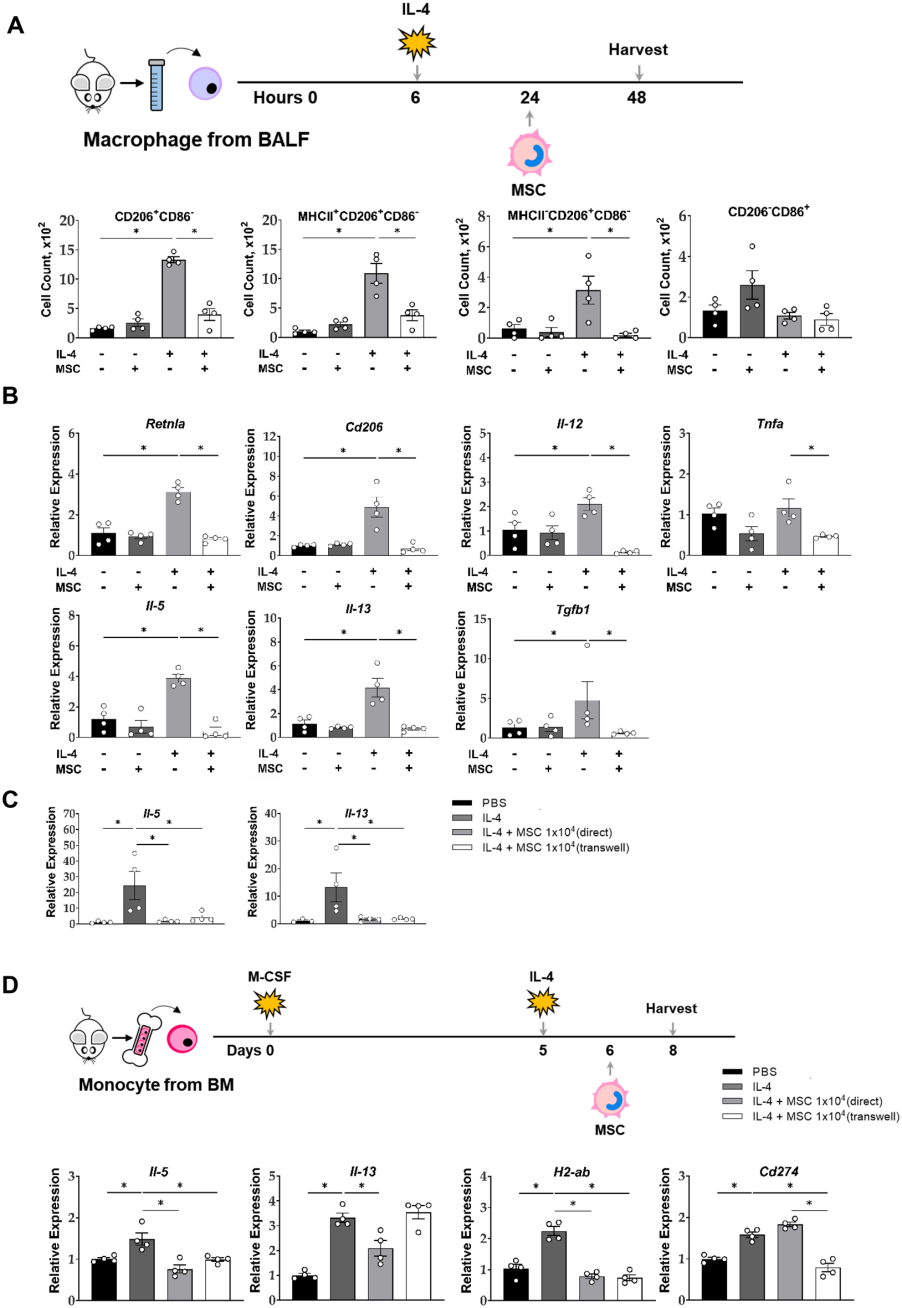
Effect of ucMSCs on mRNA expression of ex vivo and in vitro macrophages. (**A**, **B**) Changes in activation markers in the BAL fluid macrophage ex vivo. (**C**) Analysis of macrophage activation markers using alveolar macrophage cell lines (**D**) Macrophage differentiation protocol using bone marrow-derived monocytes and changes in their activation markers. *n* = 4 for each group, * indicates *P* < 0.05. All results are representative of at least three independent experiments. BAL, Bronchoalveolar lavage; MSC, mesenchymal stem cell; IL, interleukin; TNF: tumor necrosis factor.

In order to directly observe the immunomodulatory effects of ucMSCs on macrophages, bronchoalveolar lavage (BAL) fluid macrophages were collected and stimulated with IL-4 ex vivo to induce M2 macrophage polarization. In flow cytometry assays, the number of M2 macrophages and their subtypes increased upon IL-4 stimulation and was significantly improved by MSC treatment; however, there was no significant change in the M1 count (Fig. 6A). At the mRNA level, the expression of M1 markers (*Il-12* and *Tnfa*) and M2 markers (*Cd206* and *Retnla)* and Th2 cytokines (*Il-5, Il-13,* and *Tgfb1*) increased with IL-4 treatment but normalized by ucMSCs (Fig. 6B).

In addition, the CRL-2019 AM cell line stimulated with IL-4 was treated with ucMSCs to determine whether ucMSCs affected macrophages via direct (cell-to-cell contact) or indirect (transwell) mechanisms, and results show that Th2 cytokines were suppressed in both groups (Fig. 6C). Similarly, when the IL-4-stimulated bone marrow monocyte-derived macrophages were treated with ucMSCs, the mRNA expression of *Il-5* and *H2-ab* in macrophages was suppressed by both direct and indirect treatment of ucMSCs, while *Il-13* expression was inhibited by direct coculture with ucMSCs (Fig. 6D). Meanwhile, the increased expression of *Cd274* in macrophages by IL-4 stimulation was downregulated only by indirect treatment of ucMSCs using a transwell system.

## Discussion

This study successfully showed that intratracheal MSC administration modulated lung macrophages and ameliorated type 2 airway inflammation and AHR. In particular, ucMSC treatment effectively reduced the number of DCs and macrophages with antigen-presenting ability. Intratracheally instilled ucMSCs also promoted the recovery of reduced resident AMs, which in turn maintained homeostasis by suppressing inflammation^14^. Also, we found that ucMSCs affected macrophages even without direct cell-to-cell contact, suggesting that soluble factors may mediate the effect of ucMSCs. In addition, similarly with findings in our acute asthma model, 11-week chronic mouse asthma model induced by OVA challenge also showed improvement of airway inflammation and airway resistance^15^. In addition to the inflammatory changes, airway remodeling was also ameliorated, along with the reduction of lung collagen content and TGF-β1.

Stem cells are undifferentiated cells that renew via cell division and multiply into differentiated, specialized cells for various tissues and organs. MSCs are adult stem cells derived from several sources including the placenta, umbilical cord and adipose tissue, and these cells can also differentiate into various lineages^16^. A recent study confirmed that transplanted MSCs retain their immunoregulatory properties even during allogenic treatment^17,18^. Treatment with human bmMSCs influenced the development and function of immune effector cells and T-cells in mice models^19^. When bmMSCs were intravenously injected in a murine asthma model, localized bmMSCs in the lung downregulated airway inflammation and suppressed Th2 cytokines^18^. Compared to MSCs originating from the bone marrow and other adult organs^21^, ucMSCs are favorable because they can be harvested non-invasively in large amounts and most importantly, have with less ethical limitation^20^. In comparison to bmMSCs, ucMSCs have reportedly demonstrated superior immunosuppressive effects and have thus been identified as a potential treatment option for patients with severe asthma^21,22^. Until now, human-derived bmMSC studies account for the majority of MSC studies^23^. Recent studies using show that ucMSCs exhibit possible therapeutic effects by suppressing Th2 and eosinophilic inflammation^24^.

Reactive oxygen species (ROS)-mediated oxidation plays an important role in the regulation of various signal transmission molecules in MSCs and is involved in pluripotency, self-renewal capacity, and cell activity^25^. ROS can induce M2 macrophage activation in tumor-associated macrophages^26^. Glutathione (GSH) is a hydrophilic antioxidant that protects cells from ROS and reactive nitrogen species^27^. Therefore, the reduction of ROS by GSH^high^ ucMSCs might contribute to the suppression of M2 macrophages in a murine asthma model. In addition, GSH^high^ ucMSCs can enhance their therapeutic effect owing to their ability to stay longer in the lungs in vivo and relatively stronger immunomodulatory ability than GSH^low^ ucMSCs *in vitro*^28^. These findings led us to select GSH^high^ ucMSCs using FreSHtracer to potentiate the anti-asthmatic effect of MSCs.

Consistent with the findings of previous studies, the results of our studies show that ucMSC treatment reduces type 2 inflammation. The results of in vivo studies confirmed that ucMSCs decrease the number and activity of ILC2s and Th2 cells, demonstrating that ucMSCs play a role in both innate and adaptive immunity. ILC2s have been suggested as the innate counterpart of Th2 cells which play important roles in the development of asthma. The suppression of ILC2s by intravenous treatment with ucMSCs was also confirmed by using two different mouse models of severe asthma using *Alternaria alternata* and house dust mite/diesel exhaust particle^29^. Previous studies have reported that Treg upregluation may be the mechanism behind the inhibitory effects of intravenously administered ucMSCs on the Th2 response^22^. MSCs treated with IFN-γ/TNF-α showed the modulating effect on Th9 cells contributing to the development of allergy by involving ILC2 activation, IgE production, eosinophil recruitment into airway^30–33^. MSCs indirectly enhance IL-10 release by alternatively activated macrophages as well as inducing Tregs. IL-10 plays a central role of MSC-mediated regulation of the innate and adaptive immune compartments. Besides, the MSC-derived secretome has been suggested a candidate for immunomodulators. MSCs derived from Wharton’s jelly demonstrated higher IL-10 expression in the secretome than other MSCs^34^. The enzyme indoleamine 2,3-dioxygenase (IDO) suppresses lung inflammation and AHR^35^. Prostaglandin E2 (PGE2) and miRNAs (miR-155, miR-146, and miR-594) also have immunoregulatory properties^36,37^. In fact, MSC-derived IDO and PGE2 induce Tregs from naïve CD4^+^ T cells.

Moreover, the role of PD-1/PD-L1 in the immunomodulatory ability of MSCs is of interest^38^. If the function of PD-1/PD-L1 is inhibited, the induction of T cell apoptosis and the antiproliferative ability of Tregs are reduced^39–41^. On the other hand, considering the induction of M1 macrophage activation by anti-PD-L1 antibody, PD-L1 may have some roles in M2 macrophage activation^42^. Consistent with this finding, ucMSC significantly downregulated PD-L1 expression in this study. However, the detailed immunomodulatory effect of MSCs in association with immune checkpoint molecules in antigen-presenting cells, such as DCs and macrophages, needs further investigation.

If MSCs are administered intravenously, they encounter naïve CD4^+^ T cells in lymphoid organs such as lymph nodes or spleen. However, intratracheally administered MSCs are unlikely to induce Tregs because there are only few naive CD4^+^ T cells capable of differentiating into Tregs in the lungs^14^. Consequently, the lack of significant changes in the number of Treg cells in the murine asthma model following intratracheal ucMSC administration suggested that the anti-asthmatic effects of intratracheally administered ucMSCs were not mediated by Tregs. These results could be attributed to the differences in the effects of MSC administration routes. For the treatment of lung disease in a murine model, two administration routes, intratracheal and intravenous, are usually applied, but they can result in different outcomes despite treatment with the same substance, as intratracheal administration shows a local effect, while intravenous administration has a systemic effect. In terms of macrophage/monocyte systems, intratracheal administration targets lung macrophages, whereas intravenous administration exerts systemic effects by targeting the circulating monocytes. Intratracheal administration of clodronate resulted in the aggravation of asthma via depletion of AMs, whereas intravenous administration of clodronate attenuated asthma by causing the depletion of monocytes in a murine asthma model^43^. Intratracheal administration of MSCs did not induce an increase in Tregs in vivo but caused regulatory effects on lung macrophages^14^. We investigated the direct effect of ucMSCs on lung macrophage differentiation to elucidate the therapeutic effects of intratracheally administered ucMSCs. Thus, in order to elucidate an alternate mechanism behind the therapeutic effects of intratracheally administered ucMSCs, we investigated the role of ucMSCs on lung macrophage differentiation.

Macrophage activation is a dynamic process where early macrophages react to environmental signals and develop into functional macrophages^44^. When exposed to foreign substances, changes in tissue microenvironments cause macrophage polarization. In this study, the numbers of MHCII^+^CD86^+^ DCs and AMs were higher in the OVA model compared to the control group, and treatment with ucMSCs reduced these numbers. These findings suggest that ucMSCs regulate the antigen-presenting ability of DCs and AMs. In an OVA-induced murine asthma model treated with bmMSCs, the reduction in the antigen-presenting ability of DCs suggests that bmMSCs possess anti-asthmatic properties by regulating DC activation^45^.

Several studies have been conducted to functionally classify macrophages, and these studies have shown that macrophage subtypes are associated with endotypes of asthma^46–49^. An increase in M2 macrophages and the M2/M1 ratio is generally observed in patients with asthma and murine asthma models^49–52^. In particular, M2a macrophages secrete IL-5 and IL-13, which induce Th2 cell activation and initiate eosinophil infiltration in the lungs^53^. M2a macrophages expressing CD206 and MHCII increase in accordance with asthma severity, suggesting that M2a macrophages are closely involved in the pathophysiology of severe asthma^54^. Additionally, M2c macrophages have been reported to play an important role in the development of pulmonary fibrosis^55,56^, and in vitro experiments have confirmed that ASCs regulate M2c macrophage activation^57^. The results of this study showed that treating an OVA-induced asthma model with ucMSCs resulted in a change in M2 macrophage subtypes. More specifically, M2a and M2c macrophages were showed a strong positive correlation with Th2 cells, ILC2s, and eosinophils. The reduction of M2a and M2c macrophages following intratracheally administered ucMSC treatment suggests that the therapeutic effects of ucMSCs may be mediated through the regulation of these M2 macrophage subtypes.

AMs are principal immune cells that reside in the lungs and are exposed to foreign substances during the process of gas exchange. As the first line of defense against respiratory pathogens, AMs suppress inappropriate immune responses to antigens^58^. Previous studies confirmed the immunosuppressive properties of AMs by demonstrating that AM depletion leads to enhanced antigen-presenting abilities of DCs, which lead to the formation of secondary antibodies when an antigen is inhaled^59,60^. Additionally, AM-starved, OVA-sensitized mice showed an increase in eosinophilic inflammation and an enhanced Th2 response, verifying the immunosuppressive properties of AMs in the OVA-induced murine asthma model^61^.

In order to investigate the immunomodulatory role of ucMSCs on macrophages, this study analyzed the overall changes in macrophages following ucMSC treatment through CD11b and CD11c gating^62,63^. CD11b was found to aid the adhesion and migration of macrophages, helping to regulate phagocytosis and cell activation^64–66^. CD11c, the subunit that constitutes the integrin αXβ2, allows macrophages to bind to lipopolysaccharide (LPS) or act as a complement to engulf opsonized bacteria^67^. SiglecF, a marker related to eosinophil apoptosis, is expressed in eosinophils and AMs; however, this marker was not found in interstitial and inflammatory macrophages^68,69^. Although SiglecF is a marker of eosinophils, CD11c^-^ eosinophils were excluded from this study. Therefore, SiglecF^+^CD11c^+^CD11b^-^ macrophages may represent typical resident AMs with homeostatic function in a steady state. Interestingly, treatment with ucMSCs increased the number of SiglecF^+^CD11c^+^CD11b^-^ resident AMs, and this population showed a strong negative correlation with Th2 cells, ILC2s and eosinophils. These results show that ucMSCs exhibit therapeutic effects in asthma through recovery of the depleted SiglecF^+^CD11c^+^CD11b^-^ macrophage population. In accordance with the findings of this study, a previous study using OVA-induced asthma models reported that CD11c^+^CD11b^low^ AMs were significantly reduced upon OVA challenge but were restored upon treatment with human MSCs, and in vivo depletion of AMs abrogated the therapeutic effects of human MSCs on allergic airway inflammation and AHR^21^.

Recent studies confirming the immunosuppressive properties of allogenic MSCs have proposed that soluble factors may be the main mechanism behind their immunomodulatory effects^70–72^. These studies showed that immune function regulation by human MSCs is mainly attributed to immunosuppressive water-soluble agents, rather than direct cell-to-cell contact. When MSC-treated media were intratracheally administered to LPS-stimulated mice, the number of inflammatory cells in BAL fluid was decreased^70^, and other studies also suggested that MSCs mediate immunosuppressive functions by secreting water-soluble agents such as IL-6, IL-10, PGE2, and nitric oxide^71–73^.

Through ex vivo experiments, we showed that ucMSC treatment decreases the expression of M1-, M2-, and Th2-related markers. Through in vitro experiments, we found that administering ucMSCs either directly or indirectly via transwell decreased the expression of both IL-5 and IL-13. These findings are consistent with that of previous studies, and supports the idea that ucMSCs suppress type 2 inflammation by regulating macrophage activation via soluble mediators rather than direct cell-to-cell contact.

This study investigated the effects of intratracheally administered ucMSCs on lung macrophage differentiation in a murine asthma model, and our results suggest that direct delivery of ucMSCs to the airways may be a potential treatment option for severe asthma^14^. However, further studies are needed to validate the in vivo effects of MSC-induced SiglecF^+^CD11c^+^CD11b^-^ macrophages on asthmatic inflammation and identify the mechanisms by which soluble mediators of ucMSCs regulate macrophage activation. Although we focused on the anti-asthmatic effects of ucMSCs on established asthma rather than its preventive role, ucMSCc reduced the number of MHC II expressing DCs and macrophages. Therefore, measuring OVA-specific IgE, IgG1, and IgG2a can be helpful to validate whether ucMSCs play a role in the sensitization process. Finally, the xenogenic relationship between human MSCs and murine immune cells may hinder assessment of the anti-asthmatic effect. However, many studies have successfully demonstrated the anti-asthmatic effect of human MSCs using murine models, as MSCs have low immunogenicity^74–76^.

In conclusion, intratracheally administered ucMSCs inhibit AHR and type II inflammation which may be mediated by macrophage regulation in the asthmatic lung. In particular, ucMSC reduced the levels of M2a and M2c macrophages, suppressed the antigen-presenting capacity of DCs and AMs, and increased the number of resident AMs. These regulatory properties of ucMSCs show therapeutic potential in the treatment of severe asthma refractory to conventional therapy.

## Materials and methods

### Preparation of human umbilical cord-derived mesenchymal stem cells

All procedures involving human umbilical cords (hUC) or hUC-derived cells were conducted in accordance with the guidelines of the Seoul National University College of medicine. Also, all procedures, including ethics approval, proceeded with the approval of Seoul National University Hospital Institutional Review Board (IRB No. C-1708–083-878). Finally, informed consent was obtained from all subjects/participants.

hUC tissues were obtained immediately from full-term births after cesarean section. The hUCs were washed with phosphate buffered saline (PBS) to remove the vessels and amnion. The Wharton’s Jelly tissues within the hUC were isolated and minced. These explants were digested for 3h at 37℃ with an enzyme mixture (Miltenyi Biotec, Bergisch Gladbach, Germany), filtered through a 100 μM Cell strainer (BD Biosciences, Franklin Lakes, NJ, USA), and pelleted using low-speed centrifugation at 200 × *g* for 10 min. The isolated WJ-MSCs were cultured in a CellCor CD medium (Xcell Therapeutics, Seoul, Korea) supplemented with 2% human platelet lysate (hPL, STEM CELL, Vancouver, BC, Canada) in a 37°C incubator in humidified conditions with 5% carbon dioxide. The cells were harvested once they reached 90% confluency. Minimal criteria to define MSCs are; (1) plastic-adherent under standard tissue culture conditions; (2) expressing cell surface markers such as CD73, CD90, and CD105 in ≥ 95% of the MSC population and lack expression (≤ 2%) of CD11b, CD31, and CD45; (3) the capacity to differentiate into osteoblasts, adipocytes, and chondroblasts^77^.

Referring to previous studies, GSH^high^ ucMSCs were isolated from cultured ucMSCs using a fluorescent real-time thiol tracer (FreSHtracer)^77^. This tracer is a ratiometric probe capable of monitoring the ROS-induced GSH changes in living stem cells, and this process allows for the identification of cells with high GSH levels which exhibit higher therapeutic efficacy.

Finally, all the procedures were performed in accordance with relevant guidelines and regulations.

### Murine asthma model

Female 6-week-old BALB/c mice were purchased from Orient Bio (Anyang, Korea). The experiments were approved by the Institutional Animal Care and Use Committee of the Institute of Laboratory Animal Resources at Seoul National University (SNU-200302–2-2). All the procedures were performed in accordance with relevant guidelines and regulations.

Mice were sensitized with intraperitoneal injections of 100 μg OVA and 2 mg aluminum hydroxide (Sigma-Aldrich, St. Louis, MO, USA) on days 0 and 7, and the allergen challenge was performed by the intranasal injection of 50 μg OVA on days 14, 15, 16, 21, 22, and 23 (Fig. 1A).

A total of 1 × 10^5^ ucMSCs were administered intratracheally on day 17 (Fig. 1A). In order to identify changes in AHR and airway inflammation after ucMSC treatment, the mice were divided into the following four groups (*n* = 4): PBS (control group), ucMSC-treated (ucMSC group), OVA asthma (OVA group), and ucMSC-treated OVA asthma (OVA + ucMSC group). Finally, this study was performed in accordance with the ARRIVE guidelines.

### Transplantation of ucMSCs

For intratracheal administration of MSCs, the mice were anesthetized with 3% isoflurane and restrained on a board at a fixed angle. MSCs were administered to the airway using a 30-gauge needle syringe, and PBS was administered to the control group.

### Measurement of airway hyperresponsiveness and inflammation

On day 24, AHR was measured using the Buxco FinePointe Resistance and Compliance (Buxco, Wilmington, NC, USA). After the mice were anesthetized with pentobarbital sodium (50 mg/kg), measurements of lung resistance (R_L_) were carried out over 3 min. The measured R_L_ values were subtracted from the baseline values and converted to a percentage.

On the same day the R_L_ values were measured, BAL and lung excision were also performed. BAL samples were stained with Diff-Quik (Sysmex, Kobe, Japan) and cells were counted to determine the number of macrophages, neutrophils, eosinophils, and lymphocytes in each sample.

In order to identify pathological changes of the lung parenchyma, the left lung of each mouse was fixed in 2% paraformaldehyde, embedded in paraffin, and stained with hematoxylin and eosin. The degree of inflammation in histology was semiquantitatively assessed according to cell infiltration^78^.

### Lung homogenates

The lung tissues were incubated in 5 mL of RPMI1640 with Type IV collagenase (Worthington Biochemical Corporation, Lakewood, NJ, USA) at 37°C for 90min. These lung tissues were sorted through a sterile cell strainer for single cell preparation.

### Cell analysis by flow cytometry

Single-cell suspensions were treated with an FcγR-blocking monoclonal antibody (BD Bioscience). The cell surface, intracellular cytokines, and transcription factors were stained as shown in Table S1 and Fig. S1, 2.

The cells were analyzed with an LSR Fortessa X-20 (BD Biosciences) and FlowJo10 software (TreeStar, Woodburn, OR, USA). The method for the calculation of total cell number is as follows: After calculating the percentage of target cells in each tube, the ratio was multiplied by the absolute number of single cells in the lung to calculate the number of target cells in the lung. For ILC population definition (CD45^+^Lineage^-^CD90.2^+^CD4^-^), lineage markers and the CD90.2 marker were used. ILC1 and ILC3 were defined as the expression groups IFN-γ and IL-17, respectively, ILC2 was defined as the expression groups IL-5 and IL-13, and each cytokine was stained.

After eosinophils were gated out, macrophages were defined as CD45^+^F4/80^+^ cells, and DCs were defined as CD45^+^F4/80^-^CD11c^+^ cells.

M1 and M2 macrophages were defined as CD11c and CD206 single-positive cells, respectively, and M2 subtypes were further subdivided into M2a (CD206^+^MHCII^+^CD86^-^), M2b (CD206^-^MHCII^+^CD86^+)^, and M2c (CD206^+^MHCII^-^CD86^-^) subtypes^79,80^. In addition, by using CD11c and CD11b^62,63^, four clusters (CD11c^+^CD11b^-^, CD11c^+^CD11b^+^, CD11c^int^CD11b^+^, CD11c^-^CD11b^+^) were classified and analyzed (Fig. S2), and SiglecF^+^CD11c^+^CD11b^-^ were defined as resident AMs^68,69^.

The total cell calculation method is as follows. After calculating the percentage of target cells for each tube, the ratio was multiplied by the counted absolute number of single cells in the lung to calculate the number of target cells in the lung.

### ILC isolation

ILCs were isolated from single-cell suspensions obtained from mouse lungs using an EasySep Mouse Pan-ILC Enrichment Kit (STEM CELL). The isolated ILCs were treated with mouse recombinant proteins IL-2, IL-7, and IL-33 (BioLegend, San Diego, CA, USA) at 20 ng/mL for ILC2 differentiation.

### RNA isolation

The lung tissues were placed in Trizol (Thermo Fisher Scientific, Waltham, MA, USA), followed by RNA isolation using a tissue homonizer (TaKaRa, Tokyo, Japan). The RNA was isolated and reverse transcription was performed with the SensiFAS cDNA Synthesis Kit (Bioline, London, UK) according to the manufacturer’s instructions.

### Quantitative real-time PCR

Gene expression levels were measured with a 7500 real-time PCR system (Applied Biosystems, Foster City, CA, USA) after converting the extracted RNA to cDNA using a SensiMix II probe kit (Bioline, London, UK). The expression levels in each sample were normalized by *Gapdh* using the ΔΔCt method, and the relative expression level for the control sample was calculated. The following primer sequences were used and verified by PrimerBank (Harvard, MA) (Table S2).

### Ex vivo and in vitro transwell experiments

Macrophages from the BAL fluid samples were cultured in 48-well plates (1 × 10^4^ cells/well) for 6h and stimulated with 20 ng/mL of recombinant IL-4 (R&D Systems, Minneapolis, MN, USA) for 42h. Four groups were set up for ex vivo experiments: PBS (control group), ucMSC (ucMSC-treated group), IL-4 (IL-4-stimulated group), and IL-4 + ucMSC (IL-4-stimulated group treated with ucMSC). ucMSCs (1 × 10^4^ cells/well) were treated for 18h after stimulation with recombinant IL-4.

Similar to ex vivo experiments, in vitro study was conducted using the Transwell Permeable Support (Costar, Kennebunk, ME, USA). The MH-S (ATCC, Manassas, VA, USA) was cultured in 48-well plates (1 × 10^4^) and stimulated with recombinant IL-4 (R&D systems). Four groups were set up for the experiments: PBS (control group), IL-4 (IL-4-stimulated group), IL-4 + DucMSC (IL-4-stimulated group directly pretreated with ucMSC), and IL-4 + TucMSC (IL-4-stimulated group indirectly treated with ucMSC via transwell system).

### Statistical analysis

All data are expressed as means ± standard error of the mean (SEM). To analyze the correlation analysis between two groups, the Spearman's rank-order correlation test was performed. Statistical analyses were performed using GraphPad Prism 7 software (GraphPad, San Diego, CA, USA). The Mann–Whitney test was used to compare the two groups and *p*-values less than 0.05 were considered statistically significant.

## Supplementary Information

Supplementary Information.

## Data Availability

The datasets used and/or analyzed during the current study available from the corresponding author on reasonable request.
