# Supplementary material for: Intratracheal administration of mesenchymal stem cells modulates lung macrophage polarization and exerts anti-asthmatic effects
Source: Sci Rep. 2022 Jul 11;12:11728. doi: 10.1038/s41598-022-14846-y (PMC9276742; doi:10.1038/s41598-022-14846-y)
Supplement: Supplementary file 1 — Supplementary Information. [file 41598_2022_14846_MOESM1_ESM.pdf]

Intratracheal administration of mesenchymal stem cells modulates lung macrophage polarization and exerts anti-asthmatic effects.

Yosep Mo[1,2†], Hanbit Kang[1,2†], Ji-Young Bang[1,2], Jaewoo Shin[3], Hye Young Kim[1,3], Sang-Heon Cho[1,2,4], Hye-Ryun Kang[1,2,4*]

[1]*Institute of Allergy and Clinical Immunology, Seoul National University Medical Research Center, 103 Daehak-ro, Jongno-gu, Seoul 03080, Korea*

[2]*Department of Translational Medicine, Seoul National University College of Medicine, 103 Daehak-ro, Jongno-gu, Seoul 03080, Korea*

[3]*Department of Medical Science, Seoul National University College of Medicine, 103 Daehak-ro, Jongno-gu, Seoul 03080, Korea*

[4]*Department of Internal Medicine, Seoul National University College of Medicine, 103 Daehak-ro, Jongno-gu, Seoul 03080, Korea*

[†]*These authors contributed equally to this work.*

Table of Contents

Figure S1. Gating strategy for T cells and ILCs.

Figure S2. Gating strategy for macrophages.

Figure S3. Effect of ucMSCs on eosinophils and neutrophils in a murine asthma model.

Figure S4. Effect of ucMSCs on M2a and M2c by ucMSC treatment in a murine asthma model

Figure S5. Macrophage distributions based on CD11c and CD11b markers.

Table S1. Flow cytometry antibodies

Table S2. The primer sequences for RT-qPCR

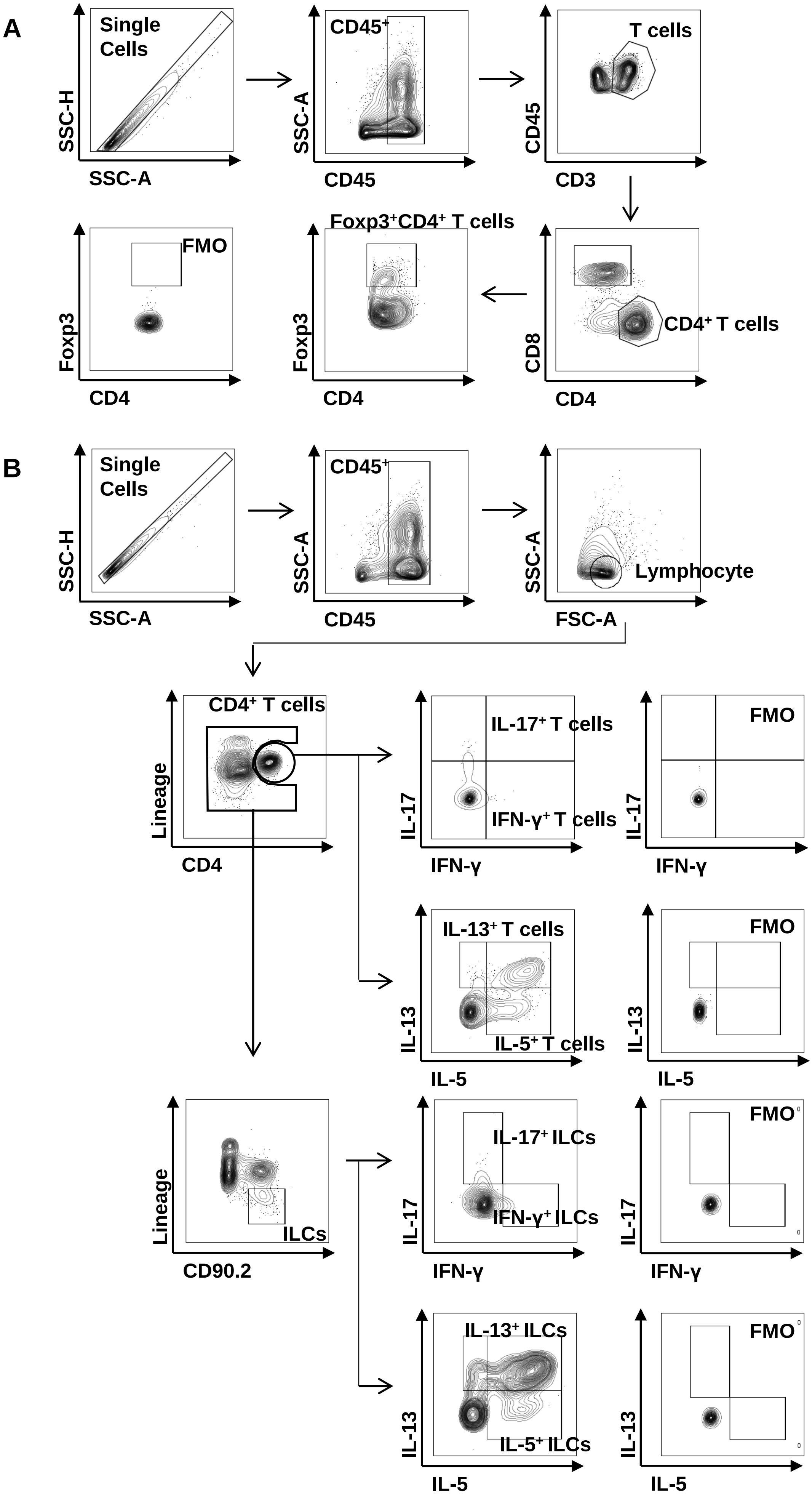
A
Single Cells
SSC-H
SSC-A
CD45$^+$
SSC-A
CD45
T cells
CD45
CD3
FMO
Foxp3
CD4
Foxp3$^+$CD4$^+$ T cells
Foxp3
CD4
CD4$^+$ T cells
CD8
CD4
B
Single Cells
SSC-H
SSC-A
CD45$^+$
SSC-A
CD45
Lymphocyte
SSC-A
FSC-A
CD4$^+$ T cells
Lineage
CD4
IL-17$^+$ T cells
IFN-γ$^+$ T cells
IL-17
IFN-γ
FMO
IL-17
IFN-γ
IL-13$^+$ T cells
IL-5$^+$ T cells
IL-13
IL-5
FMO
IL-13
IL-5
ILCs
Lineage
CD90.2
IL-17$^+$ ILCs
IFN-γ$^+$ ILCs
IL-17
IFN-γ
FMO
IL-17
IFN-γ
IL-13$^+$ ILCs
IL-5$^+$ ILCs
IL-13
IL-5
FMO
IL-13
IL-5

Figure S1. Gating strategy for T cells and ILCs. (A) Single cell suspensions prepared from OVA-induced mouse lungs were stained with antibodies against T cell markers. CD45 was used to define the lymphocyte population. After live lymphocytes were gated from CD45 cell, CD3 was used to define the total T cells. T cells were further subdivided into $CD4^+$ T cells and $CD8^+$ T cells. T-regs was then defined by gating the $Foxp3^+$ cells from the $CD4^+$ cells of CD4 versus CD8 scatter plots. (B) Similarly, CD45 was used to define the lymphocyte population. Then, $CD4^+$ cells were defined as from lineage versus $CD4^+$ T cell scatter plots and ILCs were defined from lineage versus CD90.2 scatter plots. Both effector $CD4^+$ T cells and ILCs were determined by IL-5, IL-13, IL-17, and IFN-γ positivity. Fluorescence minus one controls were used for cytokine secreting cell gating. OVA, ovalbumin; T-reg; regulatory T cell; SSC, side scatter; IL, interleukin; IFN, interferon; ILC, innate lymphoid cell.

Fig. S2A

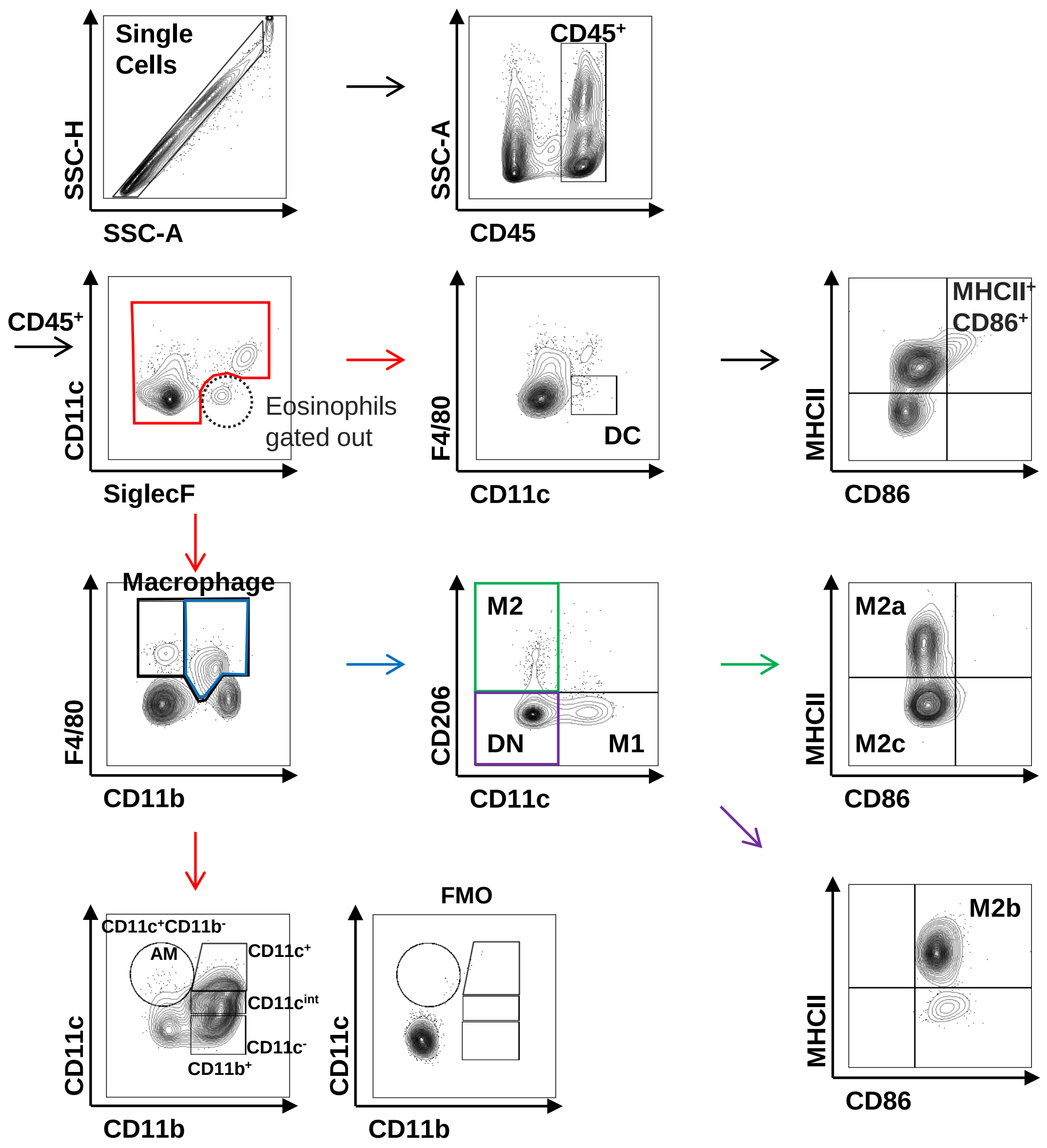

Figure S2. Gating strategy for macrophages. Single cell suspensions prepared from OVA-induced mouse lungs were stained with antibodies against macrophage markers. CD45 was used to define the lymphocyte population. After eosinophils were gated out from CD11c versus SiglecF scatter plot, F4/80 was used to define the total macrophages. Macrophages were further subdivided into DN, M1 and M2 macrophages with CD206 and CD11c. M2a, M2c, and M2b were then defined by gating on $MHCII^{+}CD86^{-}$ cells, $MHCII^{-}CD86^{-}$ cells, and $MHCII^{+}CD86^{+}$ cells from M2 and DN of CD206 versus CD11c scatter plot. Separately, in total macrophage population, using CD11c and CD11b, subtypes were divided and reanalyzed. Fluorescence minus one controls were used for gating of macrophage subtypes. SSC, side scatter; DN, double negative; DP, double positive.

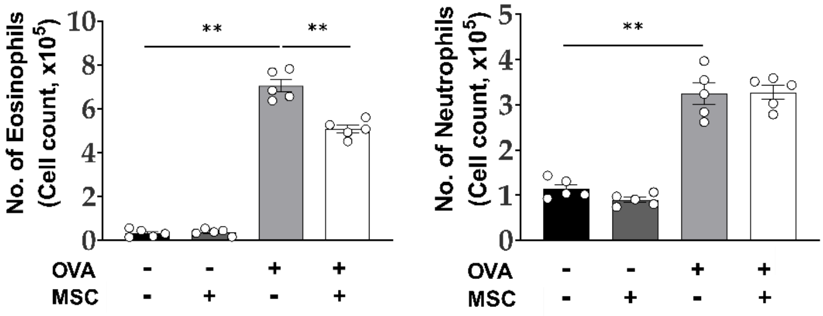

Figure S3. Effect of ucMSCs on eosinophils and neutrophils in a murine asthma model. The number of eosinophils and neutrophils. n = 5 for each group, ** indicates P < 0.01. All results are representative of at least three independent experiments. MSC, mesenchymal stem cell.

A

B

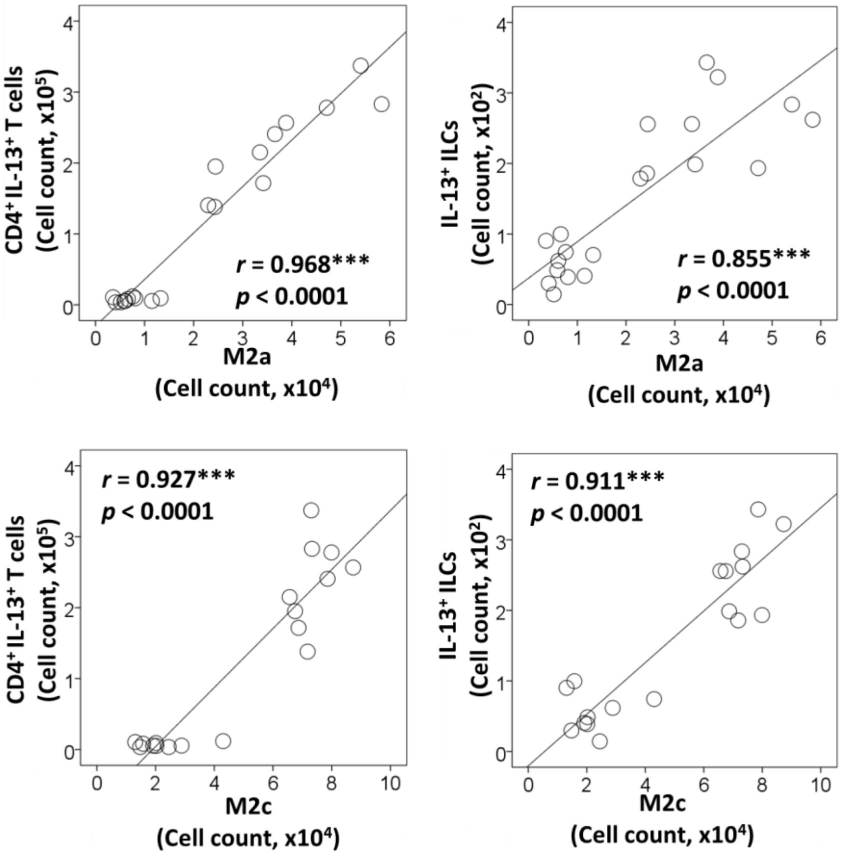

Figure S4. Effect of ucMSCs on M2a and M2c by ucMSC treatment in a murine asthma model (A) Correlation plots between M2a macrophages and $CD4^+$ IL-13^+ T cells and IL-13^+ ILCs. (B) Correlation plots between M2c macrophages and $CD4^+$ IL-13^+ T cells and IL-13^+ ILCs. n=5 for each group, *** indicates $P < 0.0001$. All results are representative of at least three independent experiments. IL, interleukin.

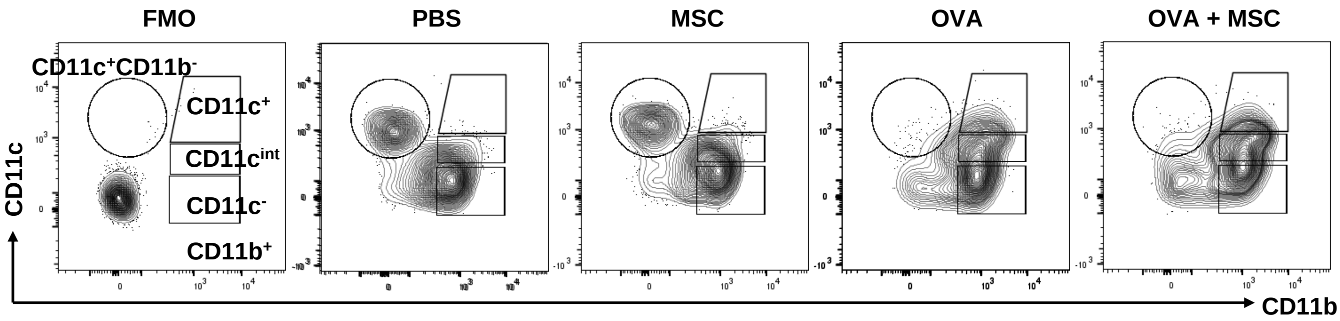

Figure S5. Macrophage distributions based on CD11c and CD11b markers. CD11c vs CD11b gating from macrophage population. PBS, phosphate buffered saline ; MSC, mesenchymal stem cell; OVA, ovalbumin.

Table S1. Flow cytometry antibodies

FcγR block staining		
Name	Clone	Company
Purified Rat Anti-Mouse CD16/CD32	Ab93	BD Biosience (BD Biosience, Franklin Lakes, NJ, USA)

Macrophage staining			
Name	Fluorescence	Clone	Company
MHCII(I-Ad)	APC	AF6-120.1	eBiosience
CD45	BV650	30-F11	Biolegend (Biolegend, San Diego, CA, USA)
SiglecF	BV421	S17007L	
Ly6c	BV510	HK1.4	
CD11c	BV711	3.9	
CD11b	BV785	M1/70	
CD86	FITC	GL-1	
F4/80	PE	BM8	
CD206	PE-Cy7	C068C2	

Lineage marker			
Name	Fluorescence	Clone	Company
CD11b	FITC	M1/70	Biolegend (Biolegend, San Diego, CA, USA)
CD11c	FITC	N418	
CD19	FITC	1D3/CD19	
CD3ε	FITC	145-2C11	
CD49b	FITC	DX5	
F4/80	FITC	BM8	
FcεRIα	FITC	MAR-1	

CD8^{+} T cell & T regulatory cell staining			
Name	Fluorescence	Clone	Company
CD45	BV650	30-F11	Bioligand
IFN-γ	APC	XMG1.2	
CD25	BV421	BC96	
CD4	BV785	RM4-5	
CD3	FITC	UCHT1	
CD8	PE	SK1	
Foxp3	PerCP-Cy5.5	R16-715	BD Biosience

CD4^{+} T cell & ILC staining			
Name	Fluorescence	Clone	Company
CD45	BV650	30-F11	Biolegend
IL-5	APC	TRFK5	
IL-17	BV421	TC11-18H10.1	
IFN-γ	PerCP-Cy5.5	4S.B3	
CD4	BV785	RM4-5	
Lineage	FITC		
IL-13	PE	W17010B	
CD90.2	PE-Cy7	53-2.1	

Table S2. The primer sequences for RT-qPCR

Table S3<br>Gene	Primer sequence
Gapdh	Forward primer: 5'-AGACTCCACGACATACTCAG-3'<br>Reverse primer: 5'-ACGGCAAATTCAACGGCACA-3'
Arg-1	Forward primer: 5'-GAATGGAAGAGTCAGTGTGGT-3'<br>Reverse primer: 5'-AGTGTTGATGTCAGTGTGAGC-3'
Retnla	Forward primer: 5'-TGCCAATCCAGCTAACTATCC-3'<br>Reverse primer: 5'-CACACCCAGTAGCAGTCATC-3'
Cd86	Forward primer: 5'-CAGACTCCTGTAGACGTGTTC-3'<br>Reverse primer: 5'-AACAGCATCTGAGATCAGCA-3'
Il-5	Forward primer: 5'-GAGATTCCCATGAGCACAGT-3'<br>Reverse primer: 5'-CTCCAATGCATAGCTGGTGAT-3'
Il-13	Forward primer: 5'-GATCTGTGTCTCTCCCTCTGA-3'<br>Reverse primer: 5'-GTCCACACTCCATACCATGC-3'
Il-12	Forward primer: 5'-CCAGGTGTCTTAGCCAGTC-3'<br>Reverse primer: 5'-CTCGTTCTTGTGTAGTTCCAG-3'
Tnfα	Forward primer: 5'-CAGGCGGTGCCTATGTCTC-3'<br>Reverse primer: 5'-CGATCACCCCGAAGTTCAGTAG-3'
Mrc-1	Forward primer: 5'-TATCTCTGTCATCCCTGTCTCT-3'<br>Reverse primer: 5'-CAAGTTGCCGTCTGAACTGA-3'
Tgfb1	Forward primer: 5'-TACCATGCCAACTTCTGTCTGGGA-3'<br>Reverse primer: 5'-ATGTTGGACAACTGCTCCACCTTG-3'
